# InCoB2014: Systems Biology update from the Asia-Pacific

**DOI:** 10.1186/1752-0509-8-S4-I1

**Published:** 2014-12-08

**Authors:** Shoba Ranganathan, Tin Wee Tan, Christian Schönbach

**Affiliations:** 1 Department of Chemistry and Biomolecular Sciences and ARC Centre of Excellence in Bioinformatics, Macquarie University, Sydney NSW 2109, Australia; 2 Department of Biochemistry, Yong Loo Lin School of Medicine, National University of Singapore, Singapore 117599; 3 Department of Biology, School of Science and Technology, Nazarbayev University, Astana 010000, Republic of Kazakhstan; 4 Center for AIDS Research, Kumamoto University, Kumamoto 860-0811, Japan

## Abstract

Selected papers from the 13th International Conference on Bioinformatics (InCoB2014), July 31-2 August, 2014 in Sydney, Australia have been compiled in this supplement. These range from network analysis and gene regulatory networks to systems level biological analysis, providing the 2014 update to InCoB's computational systems biology research.

## Introduction

Sydney, Australia hosted the 13th InCoB (International Conference on Bioinformatics), the official conference of the Asia-Pacific Bioinformatics Network (APBioNet) [1]. Since 2006, the best InCoB papers have been published as supplements in BMC Bioinformatics, with an additional supplement in BMC Genomics since 2009. With rising interest in systems biology and holistic analysis of biological data, InCoB published its first BMC Systems Biology supplement last year [2]. This introduction provides an update to the 2014 research in systems biology from the APBioNet community.

## Manuscript submission and review

Authors were offered the BMC track (supplement issues of BMC Bioinformatics, BMC Systems Biology or BMC Genomics) and PeerJ [3]. The details of acceptance rates and the reviewing process are available from our BMC Genomics overview [4]. Eleven articles with a "systems" theme briefly described in the following sections.

## Network analysis

The complexity of biological systems of often represented in terms of networks where biomolecules that interact with each other are represented as interaction partners. Characterizing a complex biological network by measuring its topology using 11 parameters and identifying nodes and sub-networks, is computationally challenging. Chin *et al.*[5] have developed cytoHubba, to analyse biological networks rapidly, rank biomolecules based on their importance and provide network visualization. This tool has been constantly improved and updated since 2009 and has been used to identify networks associated with cancer metabolism, innate immunity and even complex biofilm communities. As biological networks are dynamic, Patil and Nakai [6] have developed TimeXNet to identify temporally significant sub-networks from cellular responses to stimuli. Crosstalk between different cellular compartments is essential for for cellular communication and function. Gong and Feng [7] have analysed the ER-Golgi network and identified specific genes involved in the progress of diseases such as cancer and Alzheimer's disease. Barter*et al.*[8] have compared networks from two different cancers, to identify disease-specific biomarkers. Regulatory networks depend on relaying signals in a cascade, resulting in time delayed circuitry, which can be analysed with high levels of sensitivity with the novel method proposed by Chen *et al.*[9]. For systems and synthetic biology applications, a deep understanding of the complex flux in metabolic circuits is essential. Moriya *et al.*[10] have approached this problem by designing a synthetic circuit, as a model system for monitoring metabolic flux..

## Regulatory networks

Understanding transcriptional regulation of genes remains a challenging problem, dependent on the binding of several transcription factors as well as epigenetic changes. Given the sparseness of experimental genome-wide epigenetic profiles, Yang *et al.*[11] have developed the *cis*MEP database comprising predicted *cis*-regulatory modules integrated with available epigenetic data, as a first step to support research in this area. Approaching the transcription regulation problem from another angle, Yan and Wang [12] propose a new graph theoretical method to predict DNA binding sites from protein structures.

## Systems analysis

A comprehensive understanding of the symbiosis between the human microbiome and the host organism is essential for defining its role in human health and disease. Yang *et al.*[13] have applied an ensemble clustering framework to delineate the structure of human microbiome and provide a new insight to the pathological role of microbes within the host organism. Srihari *et al.*[14] have analysed complexes in core cellular processes to decipher cancer mechanisms, by data integration at the protein-protein interaction and gene expression levels, across all cancer conditions.

## Conclusion

The articles in this supplement span network analysis, regulatory networks as well as systems-level analysis. With ongoing NIH Big Data to Knowledge (DB2K) [15] and other similar global initiatives, we expect to see more extensive computational studies at our 2015 InCoB meeting to be held jointly with the Genome Informatics Workshop (GIW) in Tokyo, Japan [16].

## Competing interests

The authors declare that they have no competing interests.

## Authors' contributions

SR wrote the introduction. CS and SR (Program Committee Co-chairs) managed the review and editorial processes, respectively. TWT supported the post-acceptance manuscript processing.

## References

[B1] The Asia-Pacific Bioinformatics Network http://www.apbionet.org

[B2] Schönbach C Shen B Tan T Ranganathan S InCoB2013 introduces Systems Biology as a major conference theme BMC Syst Biol 2013 7 Suppl 3 S1 10.1186/1752-0509-7-S3-S1 24555777 PMC3816296

[B3] PeerJ http://peerj.com

[B4] Schönbach C Tan TW Ranganathan S Genomics 2014 15 Suppl 9 I1 25521539 10.1186/1471-2164-15-S9-I1 PMC4290585

[B5] Chin CH Chen SH Wu HH Ho CW Ko MT Lin CY cytoHubba: Identifying hub objects and sub-networks from complex interactome BMC Systems Biology 2014 8 Suppl 4 S11 25521941 10.1186/1752-0509-8-S4-S11 PMC4290687

[B6] Patil A Nakai K TimeXNet: Identifying active gene sub-networks using time-course gene expression profile BMC Systems Biology 2014 8 Suppl 4 S2 25522063 10.1186/1752-0509-8-S4-S2 PMC4290689

[B7] Gong H Feng L Computational analysis of the roles of ER-Golgi network in the cell cycle BMC Systems Biology 2014 8 Suppl 4 S3 10.1186/1752-0509-8-S4-S3 PMC4290691 25522186

[B8] Barter RL Schramm SJ Mann GJ Yang YH Network-based biomarkers enhance classical approaches to prognostic gene expression signatures BMC Systems Biology 2014 8 Suppl 4 S5 25521200 10.1186/1752-0509-8-S4-S5 PMC4290694

[B9] Chen H Mundra PA Zhao LN Lin F Zheng J Highly sensitive inference of time-delayed gene regulation by network deconvolution BMC Systems Biology 2014 8 Suppl 4 S6 25521243 10.1186/1752-0509-8-S4-S6 PMC4290726

[B10] Moriya T Yamamura M Kiga D Effects of downstream genes on synthetic genetic circuits BMC Systems Biology 2014 8 Suppl 4 S4 25521010 10.1186/1752-0509-8-S4-S4 PMC4290693

[B11] Yang TH Wang CC Hung PC Wu WS cisMEP: an integrated repository of genomic epigenetic profiles and cis-regulatory modules in *Drosophila* BMC Systems Biology 2014 8 Suppl 4 S8 25521507 10.1186/1752-0509-8-S4-S8 PMC4290730

[B12] Yan C Wang Y A graph kernel method for DNA-binding site prediction BMC Systems Biology 2014 8 Suppl 4 S10 25521807 10.1186/1752-0509-8-S4-S10 PMC4290685

[B13] Lai FJ Chang HT Huang YM Wu WS A comprehensive performance evaluation on the prediction results of existing cooperative transcription factors identification algorithms BMC Systems Biology 2014 8 Suppl 4 S9 25521604 10.1186/1752-0509-8-S4-S9 PMC4290732

[B14] Yang P Su X Ou-Yang L Chua HN Li XL Ning K Microbial community pattern detection in human body habitats via ensemble clustering framework BMC Systems Biology 2014 8 Suppl 4 S7 25521415 10.1186/1752-0509-8-S4-S7 PMC4290728

[B15] Srihari S Madhamshettiwar PB Song S Liu C Simpson T Khanna KK Ragan MA Complex-based analysis of dysregulated cellular processes in cancer BMC Systems Biology 2014 8 Suppl 4 S1 25521701 10.1186/1752-0509-8-S4-S1 PMC4290683

[B16] Big Data to Knowledge (BD2K) initiative http://bd2k.nih.gov/10.1093/jamia/ocv136 PMC5009910 26555016

[B17] GIW-InCoB 2015 http://incob.apbionet.org/incob15

